# Phormica: Photochromic Pheromone Release and Detection System for Stigmergic Coordination in Robot Swarms

**DOI:** 10.3389/frobt.2020.591402

**Published:** 2020-12-23

**Authors:** Muhammad Salman, David Garzón Ramos, Ken Hasselmann, Mauro Birattari

**Affiliations:** IRIDIA, CoDE, Université libre de Bruxelles, Brussels, Belgium

**Keywords:** collective behaviors, stigmergy, swarm robotics, indirect communication, artificial pheromone

## Abstract

Stigmergy is a form of indirect communication and coordination in which agents modify the environment to pass information to their peers. In nature, animals use stigmergy by, for example, releasing pheromone that conveys information to other members of their species. A few systems in swarm robotics research have replicated this process by introducing the concept of artificial pheromone. In this paper, we present Phormica, a system to conduct experiments in swarm robotics that enables a swarm of e-puck robots to release and detect artificial pheromone. Phormica emulates pheromone-based stigmergy thanks to the ability of robots to project UV light on the ground, which has been previously covered with a photochromic material. As a proof of concept, we test Phormica on three collective missions in which robots act collectively guided by the artificial pheromone they release and detect. Experimental results indicate that a robot swarm can effectively self-organize and act collectively by using stigmergic coordination based on the artificial pheromone provided by Phormica.

## Introduction

We present Phormica: a system that enables the realization of robot swarms that release artificial pheromone in the environment and detect it. In this paper, we demonstrate the possibility of using Phormica for realizing robot swarms that perform tasks using stigmergy to coordinate.

Stigmergy is a mechanism for agent-to-agent coordination occurring without any direct interaction between the agents. In this mechanism, each agent modifies the environment as a result of its actions and the modified environment influences the activities of the other agents (Grassé, 1959). This coordination mechanism can be observed in many social insects such as ants and termites and also in solitary insects (Wyatt, 2014; Romano et al., 2020). Insects use stigmergy to communicate about potential food sources, danger, locations of interest, or to communicate building instructions to their peers (Goss et al., 1989). The communication takes place in an indirect form: the insects use the environment as a medium to deliver messages. In the case of ants and termites, the messages take the form of trails of a chemical substance, named pheromone, that they release in the environment (Wyatt, 2014). The accumulation of pheromone stimulates the behavior of individuals of the same group: individuals that detect the trails in their close environment are likely to react to them. This process leads the group to exhibit stigmergic coordination that enables the appearance of self-organized behaviors such as foraging and aggregation, among others (Theraulaz and Bonabeau, 1999).

Pheromone is generally volatile. Indeed, a pheromone starts fading just after being deposited in the environment. Agents collectively keep releasing pheromone over time in order to reinforce a path trail or territory markers. In many cases, the agents that use this communication mechanism are state-less and do not memorize their activities. The pheromone trails in the environment serve as a shared memory mechanism. Thus, simultaneous presence, direct communication among agents, or centralized control is not necessary (Heylighen, 2016).

A robot swarm is a group of robots that operate in a distributed way and often reminiscent of the collective behavior of a colony of social insects. Robots are equipped with simple hardware and work collectively to accomplish complex missions that are beyond what can be achieved by a single robot. They typically operate in a fully decentralized manner and the individual robots do not have access to global information. Robots gather information about their environment locally through their sensors or short-range communication devices (Şahin, 2004; Dorigo et al., 2014).

A robot swarm can also leverage stigmergic coordination and exhibit several self-organized behaviors in order to perform complex missions. However, the release and detection of pheromone in robotic systems remains a difficult engineering problem.

Research work in swarm robotics has been devoted to devising different types of mechanisms for implementing artificial pheromone. Payton et al. (2001) introduced a virtual stigmergy in which pheromone is not stored in the environment but rather in neighboring robots that locally exchange messages. Virtual stigmergy has been used to conduct studies in which robots establish communication networks (Ludwig and Gini, 2006; Hamann and Wörn, 2007). Although this method relies only on local communication, the pheromone is not stored in the environment. One could argue that this method is not exactly using indirect communication to achieve stigmergy since robots rely on the presence of their peers. Other research works exist in which robots release artificial pheromone trails: trails of alcohol detected by chemical sensors (Russell, 1999; Fujisawa et al., 2014); trails of colored ink on paper (Svennebring and Koenig, 2004); trails of heat (Russell, 1997); virtual trails using an external infrastructure to record the positions of robots and project trails on the ground, or display them on a large LCD screen that acts as the floor (Hunt et al., 2019; Na et al., 2019); RFID trails where tags are installed in the environment for the robots to interact with (Khaliq et al., 2014; Khaliq and Saffiotti, 2015); and trails of phosphorescent paint (Mayet et al., 2010). Despite the importance of these approaches in the literature, we find that they have several limitations. These systems are expensive, difficult to reproduce, generally designed to execute a specific collective mission, can only be operated in certain conditions, and mostly, they are not fully autonomous—details provided in the following section.

In this paper, we introduce Phormica, a cost-effective and functional system that enables a swarm of e-puck robots to release and detect artificial pheromone in the environment. We use Phormica to conduct research in the design of collective behaviors that are based on stigmergic coordination. Phormica emulates artificial pheromone-based stigmergy. Robots operate on a surface coated with a photochromic substance that changes color from white to magenta when exposed to UV light, the magenta color fades in time and the surface turns back to white in about 50 s. Robots can deposit artificial pheromone by switching on their own UV LEDs.

Phormica has three main components: (i) the arena that is coated with the photochromic substance; (ii) the hardware add-on for the e-puck robot that is designed to emit a UV-light beam of variable size and intensity toward the ground; (iii) and the omni-directional camera with variable field of view that is used to detect the pheromone in the environment.

In order to demonstrate the capabilities of Phormica, we programmed a swarm of e-puck robots to perform three missions: (1) Coverage, (2) Foraging, and (3) Tasking.

## State of the Art

In the swarm robotics literature, researchers have adopted different approaches to implementing stigmergic coordination both in simulated and physical robot swarms. In this section, we focus our discussion only on the systems in which stigmergic coordination is achieved in a swarm of physical robots.

Payton et al. (2001) coined the term *virtual pheromones* in his well-known study titled pheromone robotics. In this study, the so-called virtual pheromone is implemented via messages locally exchanged by robots using short-range infrared communication. Robots locally broadcast messages that inform neighboring peers about the occurrence of an event or a discovery. The neighboring robots act according to the information received, or store the message based on its content, strength, or direction. These messages ultimately act as virtual agents that leave virtual pheromone on the robots (Campo et al., 2010). Many researchers have used this approach to develop communication networks, robot chain formation, and to perform foraging tasks (Maes et al., 1996; Payton et al., 2005; Ludwig and Gini, 2006). Yet, this approach uses pheromone as an analogy: pheromone is never stored in the environment and a line-of-sight between robots is necessary to pass the information.

In an alternative approach, physical memory devices are placed in an environment that acts as a shared memory for a robot swarm. For instance, a grid of radio-frequency-identification (RFID) tags is embedded into the floor on which the robots operate. Robots read and write the pheromone information on these tags using special equipment at close proximity (Khaliq et al., 2014; Khaliq and Saffiotti, 2015; Alfeo et al., 2019). However, the robots can read only one RFID tag at a time and this makes it difficult to conceive strategies based on pheromone gradients. In a related work, Allwright et al. (2014) introduced the Swarm Robotics Construction System (SRoCS). In SRoCS, stigmegy is enabled by programmable smart building blocks that are equipped with near field communication devices and can store pheromone information relevant to the construction task. The smart building blocks indicate the status of the construction by displaying different colors using RGB LEDs.

A number of researchers have proposed different approaches in which an external infrastructure is used to partially or completely store the stigmergic information. For instance, the movement of robots is tracked using an overhead camera and the pheromone trails are then either projected on the ground (Hamann et al., 2007; Garnier et al., 2013; Hunt et al., 2019; Talamali et al., 2020) or displayed on LCD screens that serve as the floor on which the robots move (Arvin et al., 2015; Na et al., 2019, 2020). The robots can detect the projected pheromone trails locally using their sensors, for then acting accordingly. These approaches are flexible in the sense that they allow displaying multiple types of pheromone at a time, and can also emulate the volatility of pheromones. On the other hand, also in these approaches, the robots are not able to autonomously perform stigmergy and depend on an external infrastructure as it is the tracking system. Moreover, these approaches are only suitable for experiments with a swarm of small robots and large-scale implementations are not feasible.

A third approach is the one in which researchers implement virtual environments to store the stigmergic information of a robot swarm. Robots can access and modify the virtual environment through virtual actuators and sensors that enable the release and detection of virtual pheromone. For example, Augmented Reality for Kilobots (ARK) is a virtual environment in which an overhead controller based on IR communication tracks the individual robots of a swarm of Kilobots, stores the pheromone information on a computer, and controls the robots accordingly (Reina et al., 2017). Similarly, Antoun et al. (2016) conceived a device named Kilogrid that enables the creation of virtual environments for swarms of Kilobots. The Kilogrid uses a floor made of a grid of communication modules that detect the position of Kilobots above them, store the information of pheromone in a virtual environment hosted in a remote computer, and controls the robots accordingly. Although, ARK and the Kilogrid are capable of executing large-scale collective mission using Kilobots, these systems are difficult to scale for a swarm of larger and faster robots.

Other studies have been conducted with techniques that enable the robots to release information markers that are detectable by the robots in physical environments. For example, odorous chemicals and alcohol are used as artificial pheromone and robots are endowed with special sensors that detect these chemicals in the environment (Russell, 1999; Fujisawa et al., 2014). Although these systems illustrate a realistic implementation of stigmergy, it remains that the use of volatile and inflammable chemicals in the environment and expensive chemical detection sensors raises questions on the practicality of the systems. Similarly, robots that carry a heating element, or hot paraffin, and leave a heat trail on the ground have been proposed as a strategy to lay down pheromone (Russell, 1997). Again, the practicality of the system is questionable. The generation of sufficient heat using the limited battery power available in many mobile robots is not a power-efficient solution and the idea of robots laying consistent heat trails for a long period is impractical.

Our research relates directly to one of Mayet et al. (2010). In their experiment, robots operate on a surface covered with phosphorescent material (glow paint) that glows in the dark when UV light is projected on it. Mayet et al. (2010) used a hardware add-on for e-puck robots to project UV light to form glow trails (pheromone trails), and additionally, used a camera to detect the glow trails in the environment. However, the system was only evaluated in simulation. The authors limited only to demonstrate the feasibility of the system with a single physical robot.

Phormica is similar to the system of Mayet et al. (2010). We use a photochromic material that changes color from white to magenta under UV light—in comparison to Mayet et al. (2010), our approach does not require a completely dark environment to display the color trails (pheromone). Our experiments go beyond the ones of Mayet et al. (2010) and comprise a swarm of e-puck robots that use Phormica to achieve stigmergic coordination in three different collective missions. In the following section, we discuss in detail the design and functionalities of Phormica.

## Photochromic Pheromone Release and Detection System (Phormica)

In this section, we discuss the construction of the artificial pheromone environment; the robotic platform; and the functionalities of Phormica.

### Artificial Pheromone Environment

**Figure 1:**
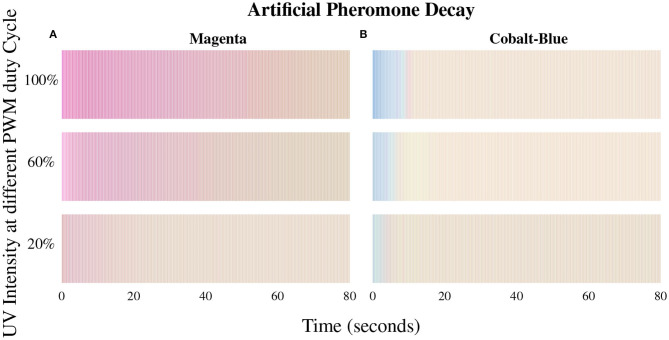
Time taken by the photochromic substances **(A)** magenta and **(B)** cobalt-blue to switch back to white color after a UV light source is removed. The substances are tested under three different intensities of UV light. The intensity of UV light is controlled by varying the pulse-width-modulation (PWM) duty cycle of UV LEDs.

In Phormica, the floor of the environment is coated with a photochromic substance that is used to store stigmergic information. Photochromic substances are a class of materials that change color when a light of specific wavelength and intensity hits them. When the light source is removed, they gradually switch back to their original color (Dürr and Bouas-Laurent, 2003). In this study, we use a commonly available photochromic substance that is of white color in its normal state and switches to magenta when lit with UV light. The color of the substance gradually decays to white after UV light is removed: this phenomenon is similar to the evaporation of biological pheromone. The saturation and decay time (pheromone evaporation) of the magenta color depends on the intensity of the incident UV light (see Figure 1). Further research is required to build the mathematical models for the color decay of these substances in the context of stigmergy for swarm robotics.

Besides magenta, this photochromic substance is also available in other colors. Substances with different colors have a different decay time and color saturation (see Figure 1). Indeed, depending on the pheromone color and evaporation time requirements, a Phormica environment can be constructed with an appropriate color of the substance.

The photochromism of this substance works fine in most indoor lighting conditions. In the case of outdoor environments, the UV light makes up a small portion of sunlight, but it is still enough to affect the photochromism in the material. The substance does not have an infinite shelf-life. Long exposures of sunlight and high temperature can alter the chemical composition of the substance. In 10 months of experiments, we never noticed any inconsistency in periods of color change cycles, but further research is required to determine the actual useful lifespan of the substance.

The photochromic substance is mostly available in pigment form. A binder is thus required to apply this substance to the floor. Depending on the application, the photochromic substance can be used with acrylics, PVC, and other resin-based binders. The type of binder and the material of the floor determine the durability of the artificial pheromone environment.

In Phormica, we use an acrylic binder with a 15% (w/w) concentration of photochromic substance. The acrylic binder has many advantages: it is low cost; easy to use; and it can be removed easily if a change of the pigment color in the same arena is needed. The technical data sheet and supplier information of photochromic substance and acrylic binder are provider as Supplementary Material (Salman et al., 2020).

The prototyping cost per square meter of the artificial pheromone environment (including 18 mm thick MDF) is approximately €25. Indeed, the most significant advantage of Phormica is the scalability of the environment: extending the size of the artificial pheromone environment only requires inexpensive materials and minimal effort.

### Robot Platform

**Figure 2:**
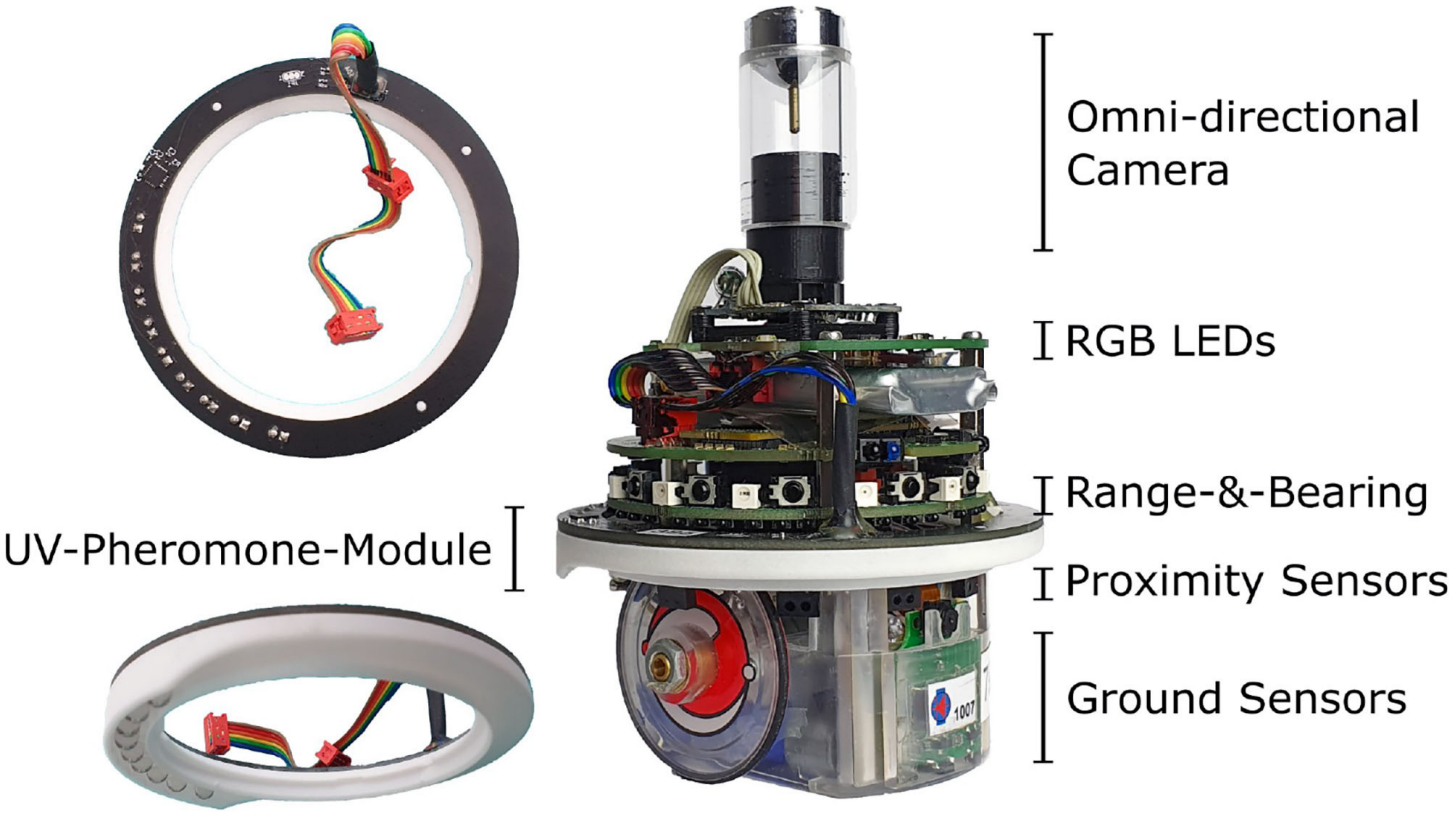
Extended version of e-puck equipped with UV-pheromone-module and omni-directional camera.

In this research, we use a swarm of e-puck robots to demonstrate the stigmergic coordination capabilities of Phormica. The e-puck robot is a differential-drive robot that is mostly used in swarm robotics research (Mondada et al., 2009). We use the extended version of the e-puck, equipped with the various computer-on-module, sensors, and communication modules: the Overo Gumstix, to run Linux on the robot; the ground sensor module to detect the gray-level color of the floor; the omni-directional camera to perceive its surroundings; and the range-and-bearing module for communication with neighboring peers (Gutiérrez et al., 2009) (see Figure 2). We formally describe the characteristics of the e-puck robot with a reference model that we shall call RM4.0 as it is a modified version of a number of previously defined reference models of the e-puck robot (Hasselmann et al., 2018; see Table 1). The reference model provides the details of the input and output variables of onboard sensors and actuators. The control software of the robot can read and/or update these variables at every control step of 100 ms. The robot can detect obstacles (*prox*_*i*_) in its surroundings using eight infrared transceivers. It can also detect the gray-level color of the floor (*ground*_*j*_) using ground sensors. The robots use the range-and-bearing module to detect the neighboring peers (*n*) within their perception range of 0.5 m, and their aggregated relative position (*V*). Three RGB LEDs, present on the robot, can display cyan or yellow color. Finally, the robot can move using two wheels whose velocity can be controlled (*v*_*r*_, *v*_*l*_).

**Table 1:** Reference Model RM4.0.

**Sensor**	**Input**	**Value**	**Description**
Proximity	*prox*_*i*∈{1, ..., 8}_	[0, 1]	Reading of proximity sensor *i*
Ground	*ground*_*j*∈{1, 2, 3}_	{*black, gray, white*}	Reading of ground sensor *j*
Range-&-Bearing	*n*	{0, ..., 4}	Number of neighboring robots perceived
	*V*	([0.5, 5];[0, 2]π rad)	Their aggregate position
Camera	*cam*_*c*∈{*R, G, B, C, M, Y*}_	{*yes, no*}	Colors perceived
	*V*_*c*∈{*R, G, B, C, M, Y*}_	(1.0;[0, 2]π rad)	Their relative aggregate direction
**Actuator**	**Output**	**Value**	**Description**
Motors	*v*_*k*∈{*l, r*}_	[−0.12, 0.12] ms^−1^	Target linear wheel velocity
RGB LEDs	*LEDs*	{ϕ, *C, Y*}	Color displayed by the LEDs
UV-pheromone-module	*phe*	({0, ..., 9}, [0, 255])	Number and brightness of UV LEDs

*The highlighted part in gray characterizes the pheromone release capabilities of the e-puck. It also represents the novelty with respect to RM3.0 (Hasselmann et al., 2018)*.

### Pheromone Release and Detection

In Phormica, pheromone release and detection is implemented using two modules: (i) the UV-pheromone-module is used to deposit the pheromone in the environment; and (ii) the omni-directional camera is used to detect pheromone in the environment.

#### UV-pheromone-module

**Figure 3:**
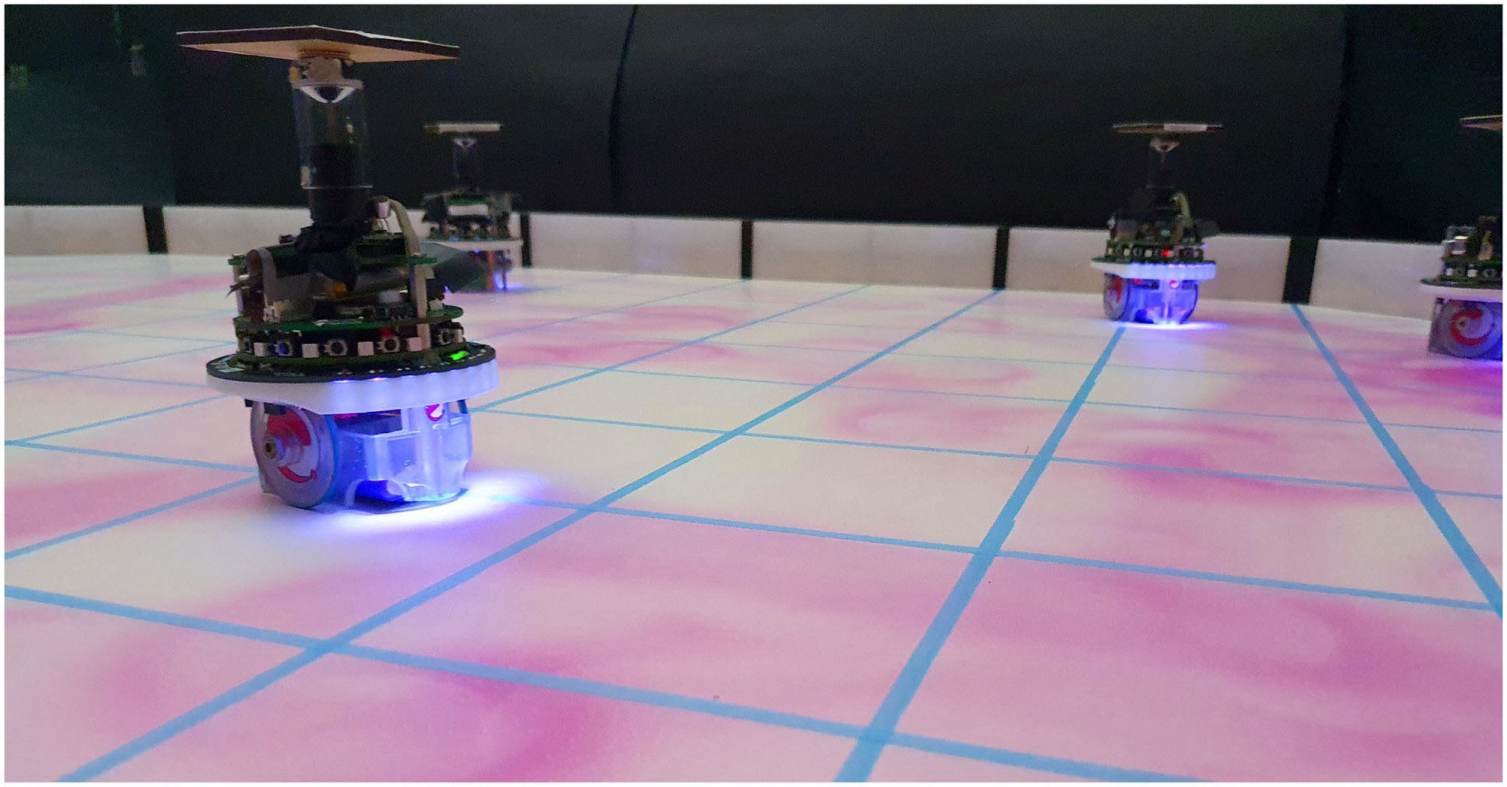
A swarm of e-puck robots equipped with UV-pheromone-module and omni-directional camera releases pheromone in the *artificial pheromone environment* to accomplish a collective mission.

In order to mimic the release of pheromone, we have developed a *UV-pheromone-module* to allow e-puck robots to project variable-intensity and variable-width UV light on the floor of the environment. The UV-pheromone-module is ring-shaped and replaces the original e-puck ring used to diffuse the light of the eight LEDs around it (see Figure 2). The UV-pheromone-module consists of two parts: (i) a printed circuit board (PCB); and (ii) a 3D-printed ring that acts as a PCB holder, gives the mechanical strength, and helps to project light uniformly on the floor (see Figure 3). The UV-pheromone-module is designed in such a way that UV light is projected only downward. A user is only exposed to the UV light if it lifts a functioning robot and turns the emitting UV LEDs toward themselves. The estimated prototyping cost of one UV-pheromone-module is €36: this price includes a 3D printed ring, a PCB, and all electronics components.

The UV-pheromone-module is equipped with nine UV LEDs facing downward and positioned at the rear of the e-puck. A nine-channel LED driver, LP55231, is used to control these LEDs. The LP55231 directly communicates with the main computer of the e-puck through I2C without using any intermediate peripheral. The LEDs are controllable separately or collectively (*phe*), to vary the intensity and beam-width of UV light (see Table 1). The intensity of the UV LEDs is controlled by a PWM (pulse-width-modulation) signal driving to LEDs. This allows the robot to control the intensity and decay time of the pheromone (see Figure 1). The UV-pheromone-module can leave a trail of pheromone of width between 30 mm, when only one LED is on, and a maximum of 75 mm, when all nine LEDs are on.

#### Omni-Directional Camera

In order to detect the artificial pheromone in the environment, the robots use an omni-directional camera. This camera can perceive red, blue, green, cyan, magenta, and yellow colors (*cam*_*c*_) in a 360 degree field of view: magenta color is reserved for the artificial pheromone—Table 1. Moreover, the field of view of the camera can be controlled to enable the robot to perceive pheromone in a given direction only: in some collective missions, it is appropriate that robots are able to differentiate pheromone released by themselves and pheromone that was already present in the environment. After perceiving the pheromone in the arena, the robot can use this information to move toward that area using a unit vector (*V*_*c*_) that represents the attraction to the pheromone perceived.

## Experimental Setup

In this section, we provide the details of the experiments in the design of collective behaviors for robot swarms that are capable of stigmergic coordination. For the purpose of this study, we use a swarm of five e-puck robots to perform experiments on three different collective missions: (1) Coverage, (2) Foraging, and (3) Tasking. For each collective mission, we perform two experiments: (i) the robot swarm performs the collective mission without using artificial pheromone; and (ii) the robot swarm takes advantage of Phormica and uses artificial pheromone.

In the three missions, the robots operate in a rectangular arena in which modular RGB blocks are placed as walls. Each RGB block is 0.25 m long and can display a color according to the mission requirements. This modular arena system was initially used by Garzón Ramos and Birattari (2020).

### Robot Control Software

We manually designed the control software of the robots—both, for the experiments in which they release pheromone and for the ones in which they do not. The control software has the form of a probabilistic finite-state machine. In this architecture, states represent low-level behaviors that the robots execute, and transition conditions represent events that trigger the change from one behavior to another. Each low-level behavior and transition condition is a parametric software module. We conceived four low-level behaviors and four transitions. The control software of the robots is obtained by configuring and assembling these software modules into finite-state machines. Table 2 describes the low-level behaviors and transition conditions we conceived for experimenting with Phormica.

**Table 2:** Low-level behaviors and transition conditions used in Phormica.

**Low-level behaviors**	**Parameters**	**Description**
Exploration	*phe*	Robot moves by random walk.
Go-to-color	c^*^, *phe, fov*	Robot steadily moves toward objects displaying a specific color.
Avoid-color	c^*^, *phe, fov*	Robot steadily moves away from objects displaying a specific color.
Waggle	τ, *phe*	Robot rotates in place for a random period of time.
**Transition conditions**	**Parameters**	**Description**
Black-floor	β	Black floor detected
Gray-floor	β	Gray floor detected
Color-detected	β, c^*^, *fov*	Objects of color perceived.
Fixed-probability	β	Transition with a fixed probability

*All low-level behaviors are capable of releasing pheromone phe (see Table 1). The parameter fov ∈ [0, 2π] determines the field of view of the camera. The parameter τ ∈ {1, .., 100} determines the number of control cycles for which a robot rotates in place: the period of the control cycle is 100 ms. In all transition conditions, the parameter β ∈ [0, 1] determines the probability of transitioning. In Phormica, the values of phe, β, and fov are determined manually by trial and error*.

* *c ∈ {R, G, B, C, M, Y}*.

The four low-level behaviors are exploration, go-to-color, avoid-color, and waggle; and the four transition conditions are black-floor, gray-floor, color-detected, and fixed-probability. In exploration, the robot moves randomly describing a ballistic motion behavior (Kegeleirs et al., 2019). Go-to-color and avoid-color are behaviors in which the robot moves toward or away from objects that display a specific color (*c*)—if the specific color is not perceived, the robot performs exploration. In waggle, the robot rotates in place for a random number of control cycles (τ ∈ {1, .., 100}). All low-level behaviors embed obstacle avoidance, and the selective release of pheromone (*phe*). The low-level behaviors and transition conditions that use the omni-directional camera to perceive color include a parameter that enables control of the field of view of the omni-directional camera (*fov*). Black-floor and gray-floor trigger a transition with a certain probability (β) if the robot detects black or gray floor, respectively. Color-detected can trigger if the robot detects a specific color (*c*). Fixed-probability triggers with a probability β.

Different combinations of these software modules into finite–state machines and different values of the parameters lead the robots to exhibit different collective behaviors. For each mission, we designed finite-state machines that enable the robots to perform the missions at hand.

### Coverage

**Figure 4:**
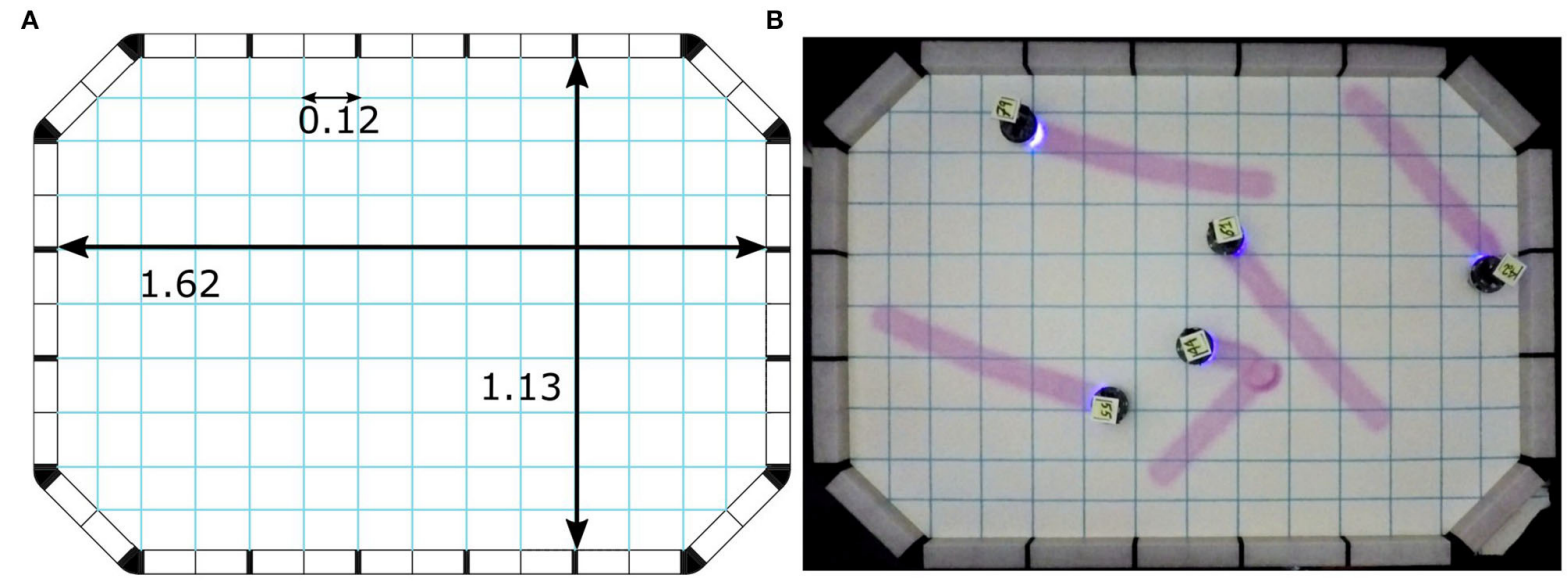
Construction of arena for the Coverage experiments: **(A)** technical representation of the arena with dimensions of mesh, and **(B)** real arena. Measurements are expressed in meters.

**Figure 5:**
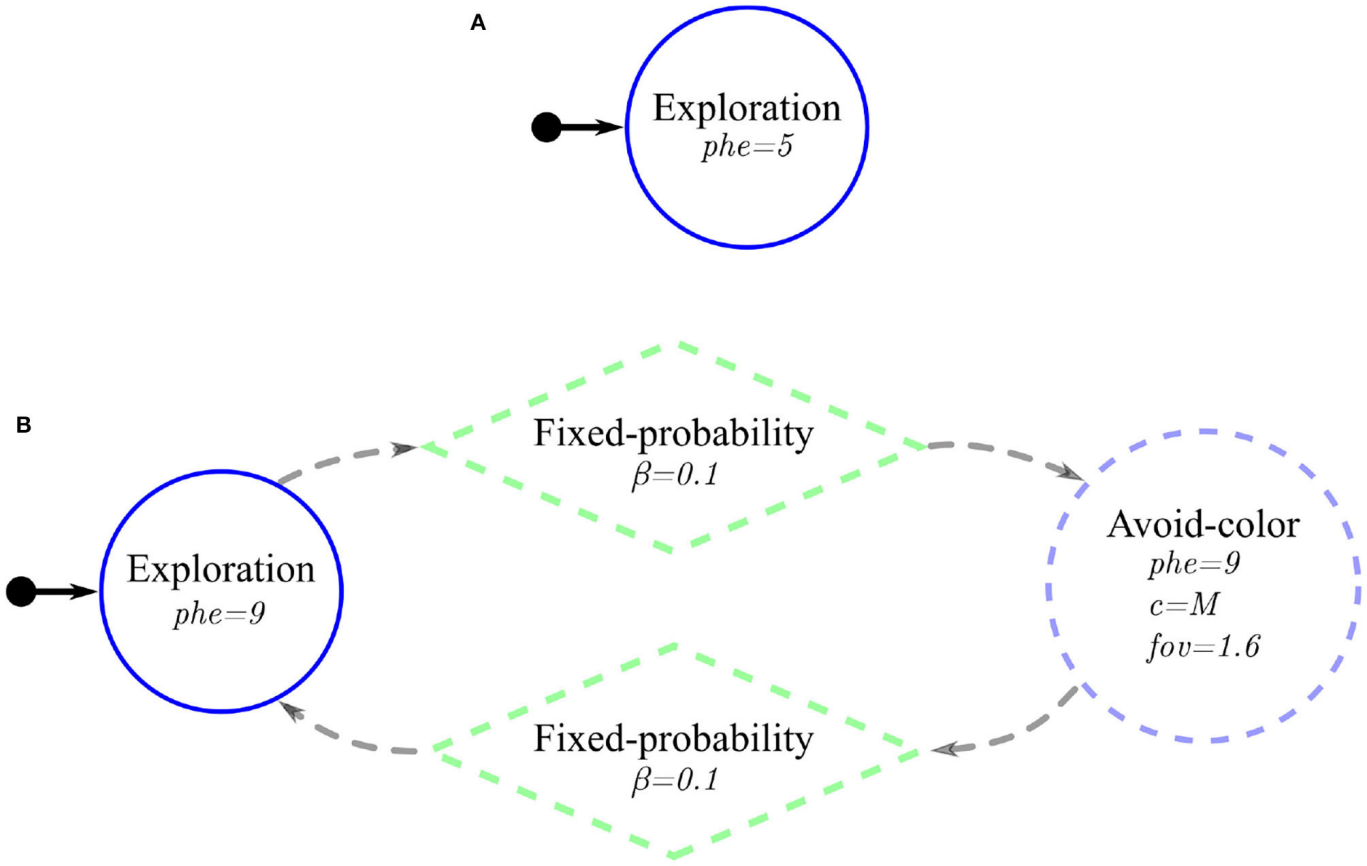
Probabilistic finite-state machines for the two Coverage experiments: **(A)** Basic-Coverage and **(B)** Phormica-Coverage—the difference between the two probabilistic finite-state machines is highlighted with dashed-lines. In both finite–state machines, a circle, a lozenge, and an arrow with a small circle represent the states, transition, and initial state, respectively.

The arena is divided into small cells to make a mesh. This mesh has 128 quadrilateral cells and eight triangular cells (see Figure 4). A robot swarm must visit each quadrilateral cell at least once in 1 min of the experiment duration. The performance *C*_*q*_ of the swarm is measured by the following objective function:

(1) $$\begin{array}{l} C_{q}=\frac{N_{c}\left(T_{cov}\right)}{Q_{c}}\times 100, \end{array}$$

where *T*_*cov*_ is the total duration of the experiment; *N*_*c*_(*T*_*cov*_) is the number of distinctive quadrilateral cells visited by the robots up to time *T*_*cov*_ = 60 s, which is the duration of a run. We perform two experiments: (i) Basic-Coverage, and (ii) Phormica-Coverage. For each experiment, we perform 20 runs of a control software in the form of a finite–state machine (see Figure 5). At the beginning of the experiments, the robots are manually placed in the arena. Their initial position and orientation is selected at random by a human experimenter.

#### Basic-Coverage

In this experiment, the robots do not employ stigmergic coordination. The robots move randomly in an attempt to traverse the whole area (see Figure 5A).

#### Phormica-Coverage

In this experiment, the robot swarm uses Phormica to achieve stigmergic coordination. The robots start a random exploration of the arena while releasing the pheromone. With a fixed probability, the robots begin to avoid previously explored areas: the robots detect the pheromone in the arena and go toward the unexplored part (without pheromone) of the arena. Similarly, with the same fixed probability, the robots again switch their behavior to random exploration (see Figure 5B).

## Foraging

**Figure 6:**
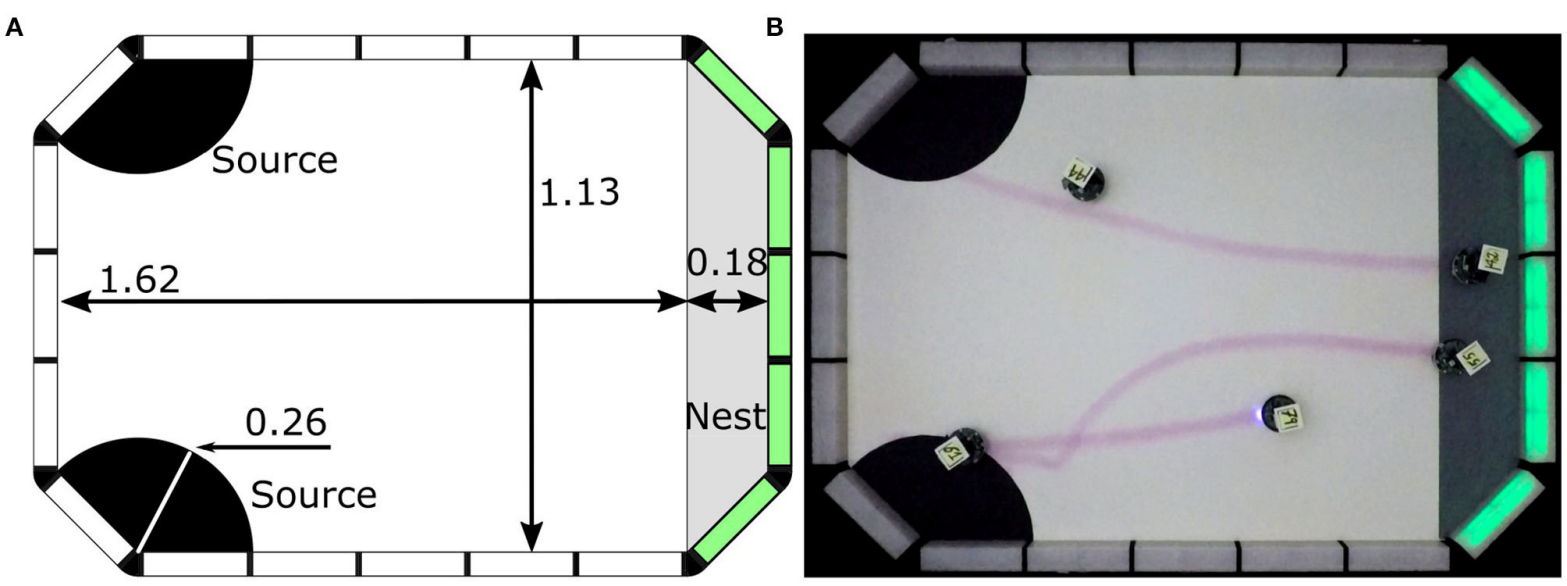
Construction of arena for the Foraging experiments: **(A)** technical representation of the arena with dimensions and positions of different zones, and **(B)** real arena. Measurements are expressed in meters.

**Figure 7:**
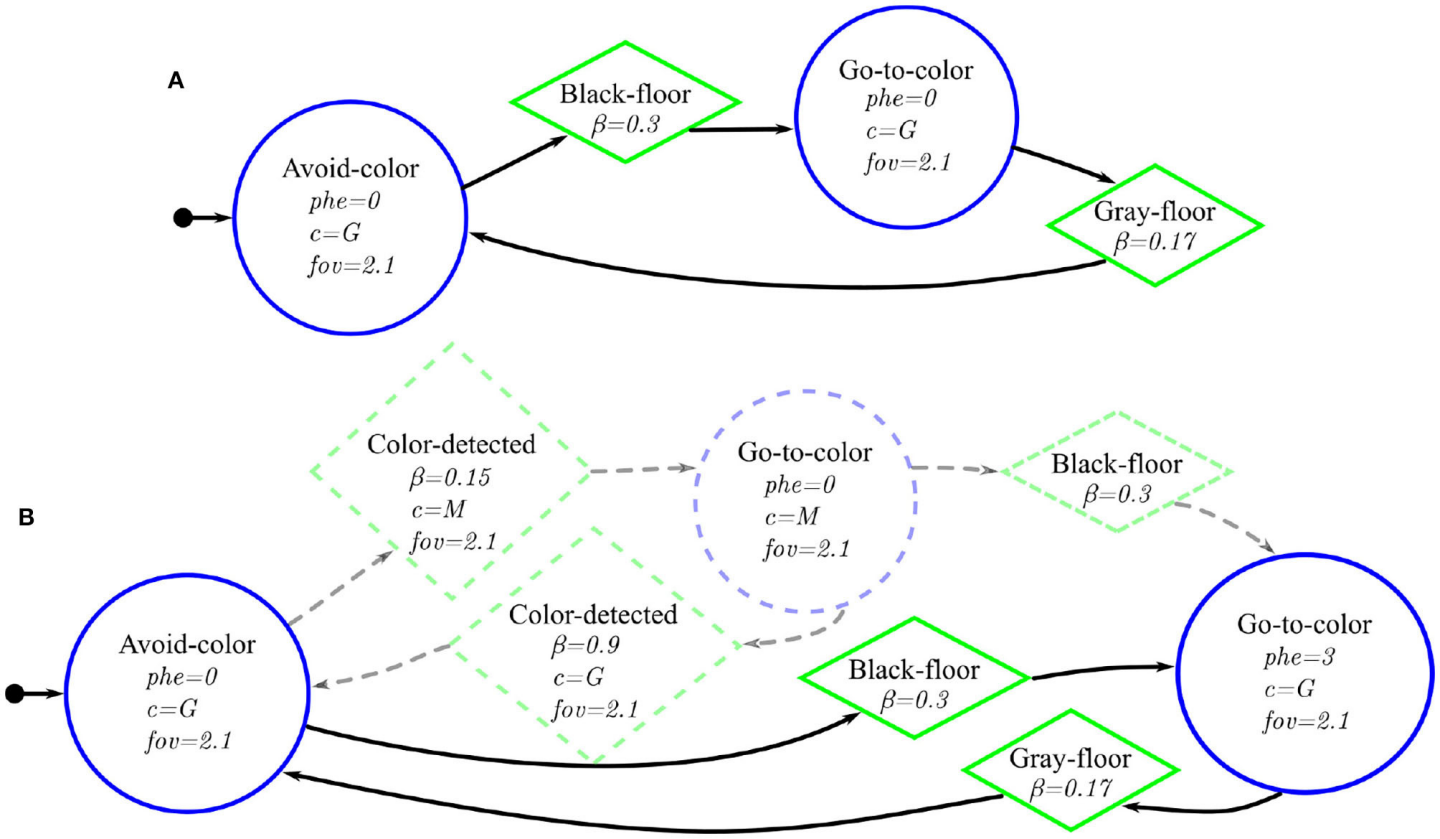
Probabilistic finite–state machines for the two Foraging experiments: **(A)** Basic-Foraging, and **(B)** Phormica-Foraging—the difference between the two probabilistic finite-state machines is highlighted with dashed-lines. In both finite–state machines, a circle, a lozenge, and an arrow with a small circle represent the states, transition, and initial state, respectively.

In Foraging, the robot swarm must collect a maximum number of objects from two sources and drop them in the nest. We abstract the Foraging experiment by considering that an object is retrieved when an individual robot visits a source, and the object is dropped when the same robot visits the nest. The two sources in the arena are represented as two black zones, while the nest is represented as a gray zone (see Figure 6). A green light is also placed behind the nest as a cue for the robots: the robots use the omni-directional camera to detect the green light (nest). The dimensions and positions of the two source zones and nest are given in Figure 6A. We perform two experiments: (i) Basic-Foraging, and (ii) Phormica-Foraging. For each experiment, we perform 20 runs of a control software in the form of a finite-state machine (see Figure 7). The performance (*F*_*F*_) of the robot swarm is computed by the following objective function:

(2) $$\begin{array}{l} F_{F}=N_{o}\left(T_{for}\right), \end{array}$$

where *T*_*for*_ is the total duration of the experiment, that is, 180 s; and *N*_*o*_(*T*_*for*_) is the number of objects retrieved by the swarm up to time *T*_*for*_. At the beginning of the experiments, the robots are manually placed in the arena. Their initial position and orientation is selected at random by a human experimenter.

### Basic-Foraging

In this experiment, the robot swarm does not use stigmergic coordination. The robots start searching for the source (black zone). When a robot reaches the source, it moves toward the nest (green light). After reaching the nest (gray zone), it again starts searching for the source. The finite–state machine that we use in this experiment is shown in Figure 7A.

### Phormica-Foraging

In this experiment, the robot swarm uses Phormica to achieve stigmergic coordination and perform foraging. The robots start searching for a source (black zone). Once a robot has reached one, it moves toward the nest (green light) while releasing pheromone. When the robot reaches the nest (gray zone), it stops releasing pheromone and follows the pheromone trail to go back to the source. The finite–state machine that we use in this experiment is shown in Figure 7B.

## Tasking

**Figure 8:**
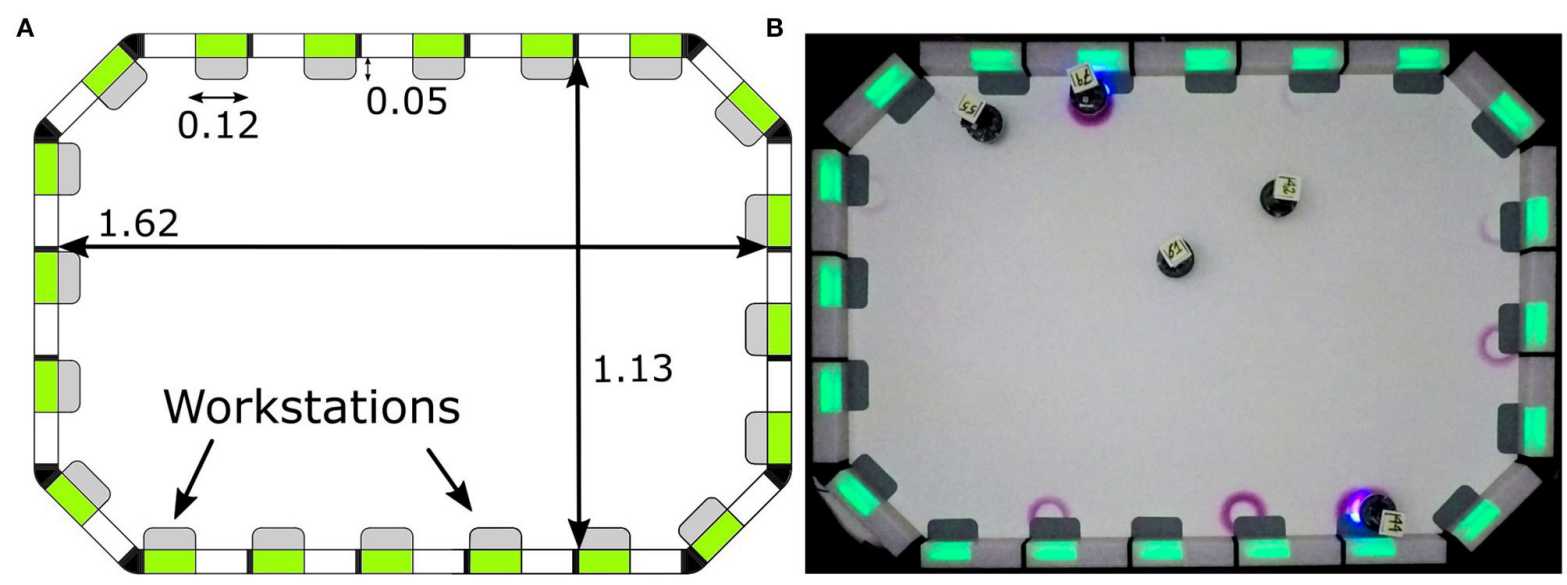
Construction of arena for the Tasking experiments: **(A)** technical representation of the arena with dimensions and position of workstations, and **(B)** real arena. Measurements are expressed in meters.

**Figure 9:**
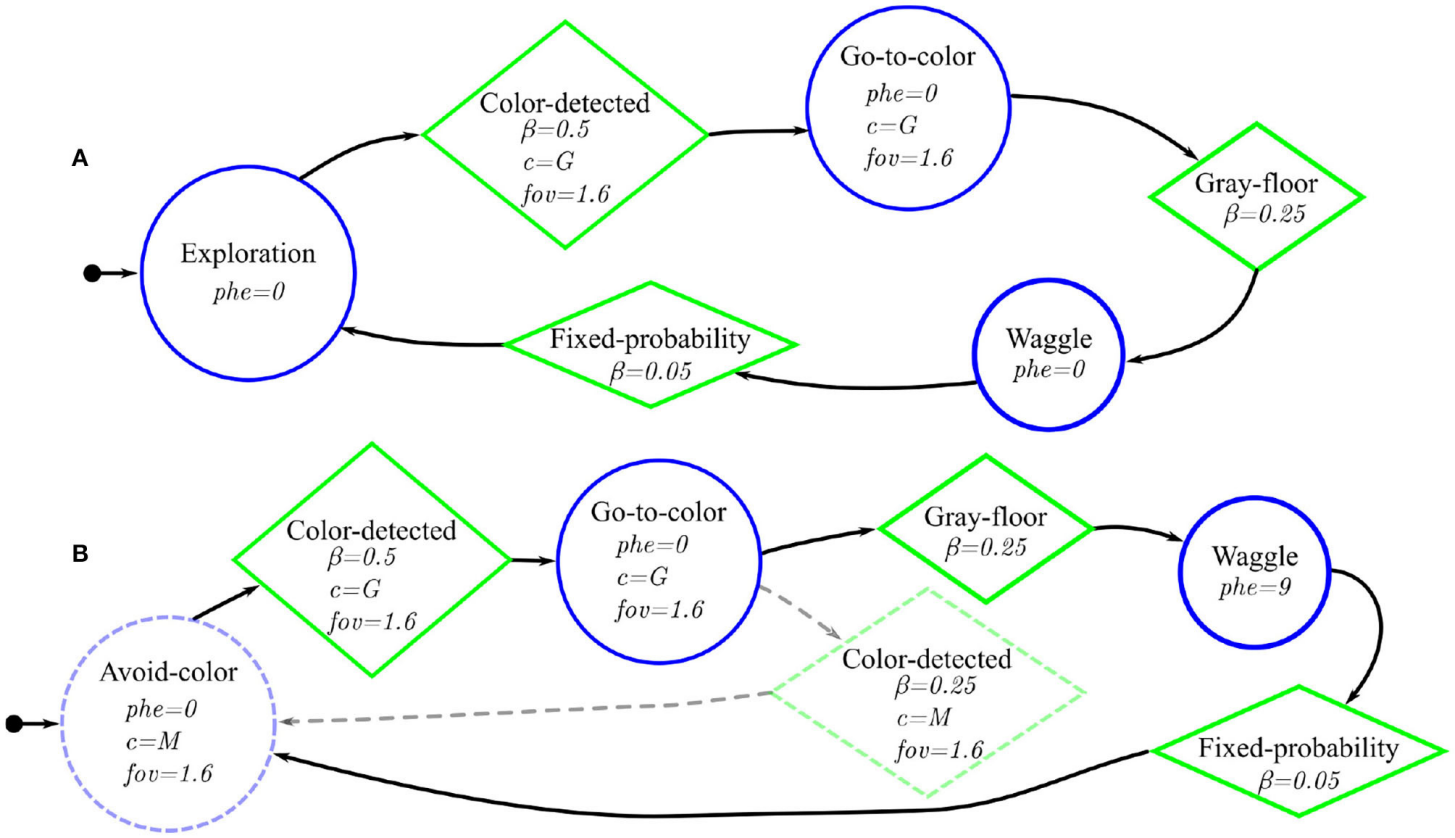
Probabilistic finite–state machines for the two Tasking experiments: **(A)** Basic-Tasking, and **(B)** Phormica-Tasking—the difference between the two probabilistic finite–state machines is highlighted with dashed-lines. In both finite–state machines, a circle, a lozenge, and an arrow with a small circle represent the states, transition, and initial state, respectively.

Tasking is a mission in which a robot swarm must perform a number of abstract tasks in a manufacturing facility. Robots perform a task in workstations that are indicated by gray patches on the floor and LED blocks displaying the color green. The manufacturing facility has 20 workstations (see Figure 8). The dimensions and positions of the workstations are given in Figure 8A. We consider that a task is performed when a robot steps into the corresponding workstation and spins on its axis (waggle). The performance of the robot swarm is measured by the number of tasks performed on distinct workstations in 2 min. The robots must visit a workstation only once. Robots receive a penalty if they perform a task on a workstation more than once. The performance *T*_*p*_ of the robot swarm is computed by the following objective function:

(3) $$\begin{array}{l} T_{P}=T_{d}\left(T_{tas}\right)-T_{rep}\left(T_{tas}\right), \end{array}$$

where *T*_*tas*_ is the total duration of the experiment, that is, 120 s; *T*_*d*_(*T*_*tas*_) is the number of tasks performed on distinct workstations; and *T*_*rep*_(*T*_*tas*_) is the number of tasks performed on a workstation more than once up to time *T*_*tas*_. We perform two experiments: (i) Basic-Tasking, and (ii) Phormica-Tasking. For each experiment, we perform 20 runs of a control software in the form of a finite–state machine (see Figure 9). At the beginning of the experiments, the robots are manually placed in the arena. Their initial position and orientation is selected at random by a human experimenter.

### Basic-Tasking

The robots start searching for a workstation. Once a robot arrives at a workstation, it waggles at the spot. As the robots do not use the UV-pheromone-module, they are unable to mark the task and hence cannot identify the workstations that have been already served. The finite-state machine that we use in this experiment is shown in Figure 9A.

### Phormica-Tasking

In this experiment, the robot swarm uses Phormica to release and detect artificial pheromone at the workstations as an indication of a completed task. The robots look for workstations to perform available tasks. If a robot perceives a pheromone indication, it avoids that workstation and looks for another one. Once a robot arrives at a workstation that has no pheromone, it waggles at the spot and releases pheromone to indicate that a task was already performed there. The finite-state machine that we use in this experiment is shown in Figure 9B.

## Results

In this section, we present the results on a per-mission basis. The performance scores of experiments are evaluated by manually post-processing the experiment videos^1^. We use box-plots to compare the performance observed when Phormica is used and when it is not (see section Experimental Setup). The control software, the data collected, and videos of the experiments are available as online Supplementary Material (Salman et al., 2020).

### Coverage

**Figure 10:**
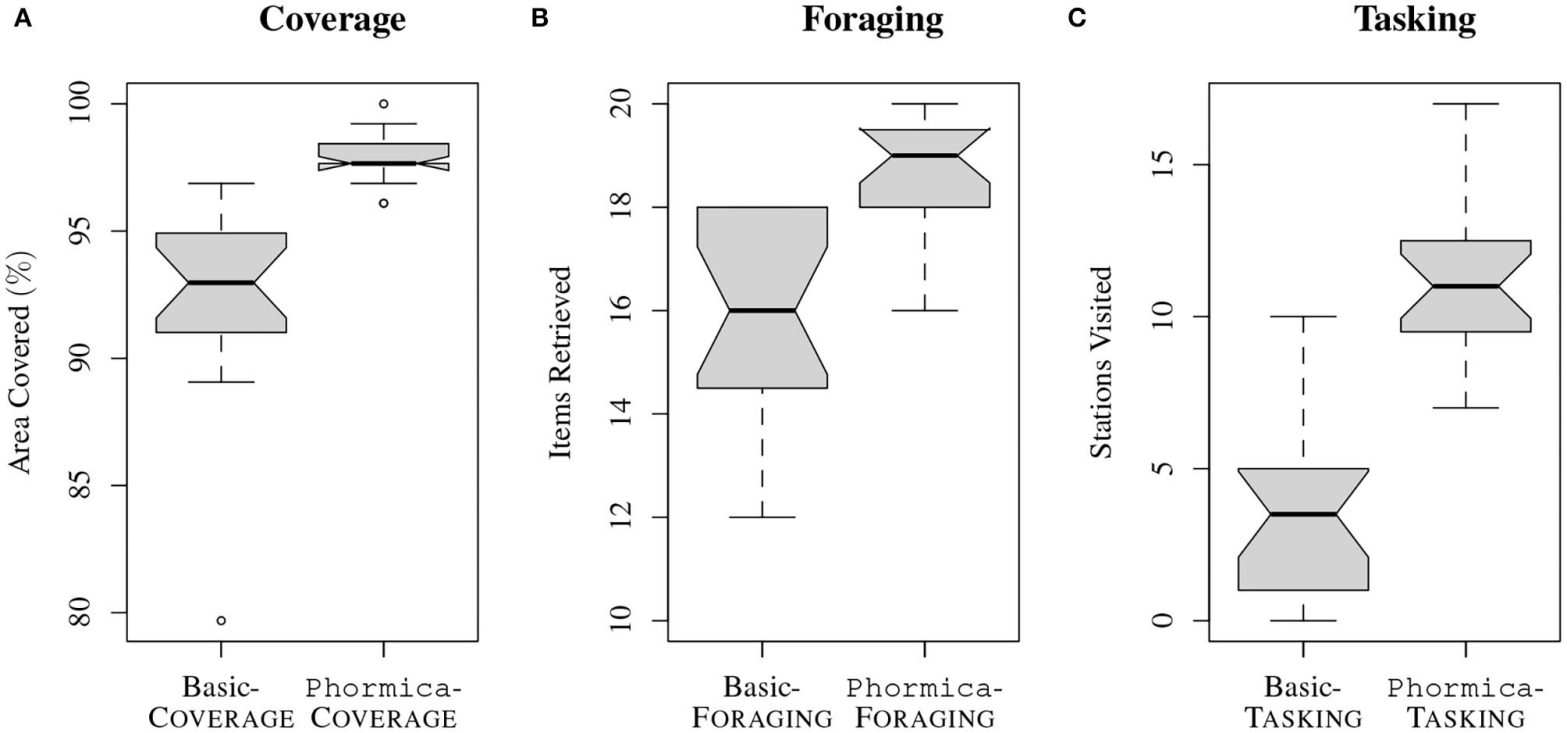
Performance obtained in the missions **(A)** Coverage, **(B)** Foraging, and **(C)** Tasking.

In Basic-Coverage, the robots are unable to differentiate between the explored and unexplored regions of the arena. Therefore, many parts of the arena remain unexplored: the robots waste time re-exploring the already visited ones. On the other hand, in Phormica-Coverage, the robots use Phormica to achieve stigmergic coordination that enables them to avoid revisiting the already explored parts of the arena. Consequently, in Phormica-Coverage the swarm explores a significantly wider area than in Basic-Coverage (Wilcoxon signed–rank test, confidence 95%) (see Figure 10A).

**Figure 11:**
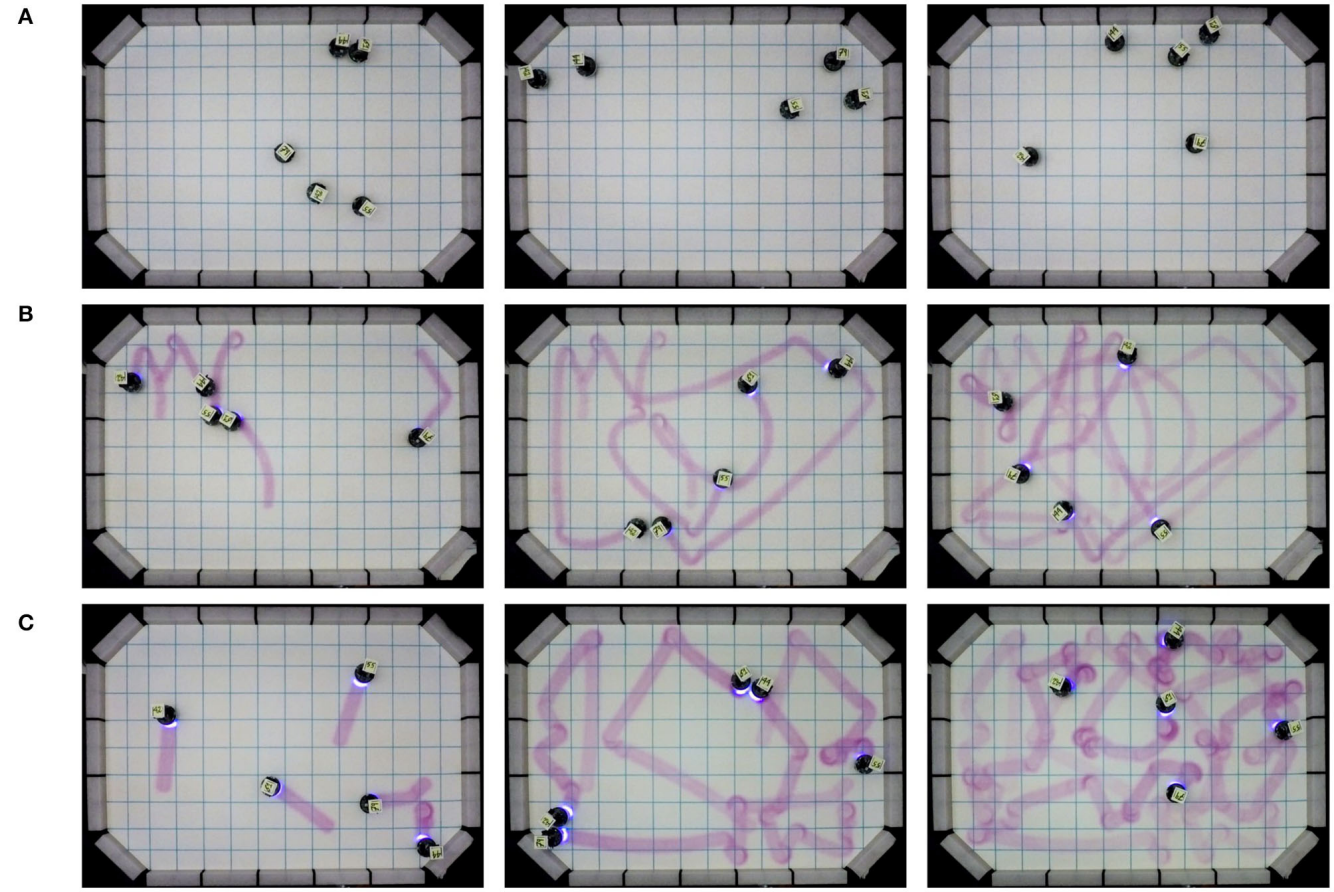
Motion patterns emerged during different Coverage experiments. **(A)** e-puck robots performing Basic-Coverage: robots tracking is impossible without an external infrastructure. **(B)** Robots performing Basic-Coverage, they use Phormica only to release pheromone. **(C)** Robots performing Phormica-Coverage: they release pheromone and are also stimulated by its presence.

We observe that the visual assessment of the performance of Basic-Coverage is particularly difficult without an external infrastructure—i.e., a tracking system (see Figure 11A). In order to make Basic-Coverage experiments visually comprehensible, the robots turn on their UV LEDs to leave the trails of pheromone while doing random walk: these trails do not stimulate any behavior in robots (see Figure 11B). Indeed, the pheromone release feature of Phormica can also be used to visualize the motion of individual robots of a swarm and visualizing the area covered by a robot swarm (see Figure 11).

### Foraging

**Figure 12:**
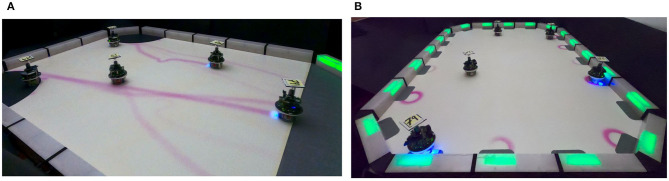
**(A)** A swarm of e-puck robots depositing pheromone trails from source to nest using Phormica to perform Foraging, and **(B)** a swarm of e-puck robots using Phormica to mark the workstations where they perform a task.

In Basic-Foraging, the robots are not aware of the location of the source in the arena. On the other hand, in Phormica-Foraging, the robots deposit pheromone trails from source to nest that other robots (or same robot) use them to track the location of the source: the robots spend less time searching for the source compared to Basic-Foraging (see Figure 12A). The results show that in Phormica-Foraging the robot swarm retrieved significantly more items than in Basic-Foraging (Wilcoxon signed–rank test, confidence 95%) (see Figure 10B).

### Tasking

In Basic-Tasking, the robots do not use pheromone markers on workstations and are therefore prone to visit a workstation more than once. On the contrary, in Phormica-Tasking, the robots deposit pheromone to differentiate whether a workstation is available or a task has already been performed (see Figure 12B). As expected, results show that in Phormica-Tasking the robot swarm obtains a significantly higher score than in Basic-Tasking (Wilcoxon signed–rank test, confidence 95%) (see Figure 10C).

## Discussion

In this section, we discuss in detail the current limitations and possible extensions of Phormica; what can be immediately done to enhance its usability; and the prospects of this approach.

In Phormica, the pheromone release and detection system is developed for e-puck robots. Indeed, this system can be implemented for any robot platform that has at least the capability to detect colors in the environment. However, if only the visualization of motion patterns during a collective mission is required, this system can easily be implemented for most of the small robots presently being used in swarm robotics research (Nedjah and Junior, 2019). Due to the effectiveness and flexibility of this approach, research communities other than the one of swarm robotics, for example biologists, might also benefit from this approach.

As described earlier, the ground in Phormica is painted with a photochromic substance that changes color from white to magenta when exposed to the UV light. This process is reversible: the color of the substance changes back to white when UV light is removed. However, UV exposure for an extended period can damage the molecular structure of the substance and might reduce its life span: it might no longer exhibit reversible photochromism. Indeed, the time this substance needs to return to white (color-decay-rate) is analogous to the evaporation time of naturally occurring pheromone. However, the formal mathematical models to determine the color-decay-rate of this substance are not available. Therefore, further study with more focus on determining color-decay-rates of all available colors of this type of photochromic substance is therefore suggested.

This substance is usually available in pigment form and could be used to produce tiles or sheets that would facilitate the creation of environments in which robot swarms could operate relying on stigmergic coordination. These tiles or sheets could be installed, for example, in a factory or office floor to enable stigmergic coordination in commercial environments. This could open the possibility of interaction between robots and humans: for instance, robots could draw a line on the floor or wall that humans can follow; and humans could also leave similar markers to interact with the robots.

The research in the field of material sciences is evolving rapidly and we might expect that, in the future, photochromic materials with a longer life span might be developed. We can also expect that a similar photochromic substance could become available that displays different colors depending on the wavelengths of the light to which it is exposed. Robots could then be able to leave or draw different color markers to communicate different messages to humans and other robots.

## Conclusion

In this paper, we presented a cost-effective and functional system, Phormica, that enhances the capabilities of a swarm of e-puck robots and enables them to release and detect artificial pheromone in the environment. Phormica is based on a photochromic substance that emulates artificial pheromone when exposed to UV light. We demonstrated Phormica on three collective missions that benefit from stigmergic coordination. The results of our experiments indicated that robot swarms that take advantage of Phormica to achieve stigmergic coordination perform significantly better than the swarm that relies on other collective behaviors to accomplish the mission. Besides enabling stigmergic coordination, the technology we proposed in the paper can also be used as a visualization tool to observe motion patterns, various random walks, or other behaviors without the help of any complex external infrastructure. The outcomes of this research demonstrate the significance and usability of Phormica in swarm robotics research.

## Data Availability Statement

The datasets generated for this research can be found in the IRIDIA Supplementary Information (ISSN: 354 2684-2041) at: http://iridia.ulb.ac.be/supp/IridiaSupp2020-010/.

## Author Contributions

All authors contributed to the elaboration of the ideas presented in the paper, read the text, and provided comments. In particular, MS implemented the system and conducted the experiments. DGR contributed to the design of software components. The paper was drafted by MS and refined by all authors. The research was directed by MB.

## Conflict of Interest

The authors declare that the research was conducted in the absence of any commercial or financial relationships that could be construed as a potential conflict of interest.

## Footnotes

^1^The experiments were originally planned to be executed in a well-equipped robotics experiment facility that is also equipped with a tracking system. But due to COVID-19 confinement measures, we were forced to move the experimental setup to MS's residence where it was unfeasible to set up a tracking system from scratch. Although we evaluated the performance manually, we are confident that our evaluation is accurate and that the results we obtained can be reproduced.
